# Comparing Ultra-hypofractionated Proton versus Photon Therapy in Extremity Soft Tissue Sarcoma

**DOI:** 10.14338/IJPT-22-00022.1

**Published:** 2023-01-16

**Authors:** Rehema Thomas, Hao Chen, Emile Gogineni, Aditya Halthore, Bethlehem Floreza, Temiloluwa Esho-Voltaire, Arcelia Weaver, Sara Alcorn, Matthew Ladra, Heng Li, Curtiland Deville

**Affiliations:** 1 Department of Medicine, Memorial Sloan Kettering Cancer Center, New York, NY, USA; 2 Department of Radiation Oncology and Molecular Radiation Sciences, Johns Hopkins University School of Medicine, Baltimore, MD, USA; 3 Department of Radiation Oncology, University of Minnesota Medical School, Minneapolis, MN, USA

**Keywords:** soft tissue sarcoma, proton therapy, hypofractionation, ultra-hypofractionation, stereotactic body radiation therapy, stereotactic body proton therapy, preoperative radiation, neoadjuvant radiation

## Abstract

**Purpose:**

Recent single institution, phase II evidence has demonstrated the feasibility and efficacy of ultra-hypofractionated, preoperative photon therapy in 5 fractions for the treatment of soft tissue sarcoma (STS). Our purpose was to evaluate the dosimetric benefits of modern scanning beam proton therapy compared with conventional photon radiation therapy (RT) for the neoadjuvant treatment of adult extremity STS.

**Materials and Methods:**

Existing proton and photon plans for 11 adult patients with STS of the lower extremities previously treated preoperatively with neoadjuvant RT at our center were used to create proton therapy plans using Raystation Treatment Planning System v10.A. Volumes were delineated, and doses reported consistent with International Commission on Radiation Units and Measurements reports 50, 62, and 78. Target volumes were optimized such that 100% clinical target volume (CTV) was covered by 99% of the prescription dose. The prescribed dose was 30 Gy for PT and RT delivered in 5 fractions. For proton therapy, doses are reported in GyRBE = 1.1 Gy. The constraints for adjacent organs at risk (OARs) within 1 cm of the CTV were the following: femur V30Gy ≤ 50%, joint V30Gy < 50%, femoral head V30Gy ≤ 5 cm^3^, strip V12 ≤ 10%, and skin V12 < 50%. Target coverage goals, OAR constraints, and integral dose were compared by Student *t* test with *P* < .05 significance.

**Results:**

A minimum 99% CTV coverage was achieved for all plans. OAR dose constraints were achieved for all proton and photon plans; however, mean doses to the femur (10.7 ± 8.5 vs 16.1 ± 7.7 GyRBE), femoral head (2.0 ± 4.4 vs 3.6 ± 6.4 GyRBE), and proximal joint (1.8 ± 2.4 vs 3.5 ± 4.4 GyRBE) were all significantly lower with PT vs intensity-modulated radiation therapy (IMRT) (all *P* < .05). Integral dose was significantly reduced for proton vs photon plans. Conformity and heterogeneity indices were significantly better for proton therapy.

**Conclusion:**

Proton therapy maintained target coverage while significantly reducing integral and mean doses to the proximal organs at risk compared with RT. Further prospective investigation is warranted to validate these findings and potential benefit in the management of adult STS.

## Introduction

Sarcomas are a heterogeneous category of cancers occurring in the bone and soft and connective tissues throughout the body [1]. An estimated 13 190 new cases of soft tissue sarcoma and 5130 deaths are expected in the United States in 2022 [2]. Limb salvage surgery and radiation therapy (RT) form the basis of extremity soft tissue sarcoma management. Amputation is generally reserved for salvage treatment. This combined modality treatment strategy is based on randomized evidence demonstrating comparable outcomes to upfront amputation [3–5].The rates of local control following surgery and radiation approach 90% to 100% for low-grade and 85% to 90% for high-grade extremity soft tissue sarcoma, respectively [6]. However, wound healing complications and late fibrosis remain consequences of this treatment approach, with the former being the predominant toxicity associated with preoperative RT despite efforts to reduce acute toxicities in the image-guided radiotherapy (IGRT) era [7–9]. In a trial by O'Sullivan et al, patients with tumors of the proximal lower extremity (ie, thigh) treated with preoperative RT experienced the highest rate of wound complications (proximal lower extremity 45%, distal lower extremity 38%, proximal upper extremity 10%) [8].

Ultra-hypofractionated preoperative RT has been shown to provide acceptable outcomes in retrospective studies and prospective trials and has several potential advantages over conventional fractionation, such as patient convenience, shorter interval from diagnosis to definitive surgical intervention, and radiobiological benefit. Radiobiological improvement in the therapeutic ratio owing to the low α/β of soft tissue sarcoma suggests a potential benefit to using higher doses per fraction. A recent phase II trial evaluating the use of preoperative radiotherapy to 30 Gy in 5 fractions for patients with high-risk primary soft tissue sarcoma found favorable rates of grade ≥2 radiation-associated toxicity (16%) after median follow-up of 2 years, with a 32% rate of wound healing complications [10]. Other institutional experiences have similarly reported 15% to 35% rates of late grade ≥2 toxicity and 25% to 40% rate of wound complications associated with preoperative ultra-hypofractionated RT, as shown in **Table 1** [10–13].

**Table 1.** Reported prospective studies of hypofractionated preoperative photon therapy for soft tissue sarcoma.

Study	N	Dose/fraction and frequency	BED (Gy)^a^	Radiation technique	Time to surgery	Chemotherapy (%)	R0 Resection rate (%)	Local control	Toxicity^b^	Wound complications
Kosela-Paterczyk et al, 2014 [11]	272	25 Gy/5 fx Daily	56	3DCRT	3-7 d	22	79	3 y: 81%	late g2 15%	32% (7% requiring reoperation)
Kubicek et al, 2022 [12]	15	35-40 Gy/5 fx (median 35 Gy) Every other day	96-120 (median 96)	SBRT (cyberknife)	29-83 d (median 41)	19	80	4.7 y: 93%	late g1-2: 27% late g4: 7%	20%
Kalbasi et al, 2020 [10]	52	30 Gy/5 fx Daily	75	IMRT: 76% 3DCRT: 20% Electrons: 4%	2-6 wk (median 4 wk)	12	82	2 y: 94%	late g2: 16%	32% requiring reoperation
Bedi et al, 2022 [13]	32	35 Gy/5 fx Every other day	96	3DCRT or IMRT	19-67 d (median 41 d)	31	91	3 y: 100%	Fibrosis g2: 22% g3: 13%	25%

**Abbreviations:** BED, biologically effective dose; R0, microscopically margin-negative resection, in which no gross or microscopic tumor remains in the primary tumor bed; fx, fraction; 3DCRT, 3-dimensional conformal radiotherapy; SBRT, stereotactic body radiation therapy; IMRT, intensity-modulated radiation therapy.

a Assuming α/β of 4 Gy.

b g1, g2, g3, and g4 indicate grades 1, 2, 3, and 4 toxicity, respectively.

Owing to the physical properties of the beam, specifically the Bragg peak and lack of exit dose, proton therapy is generally associated with a dosimetric benefit in comparison with photon therapy. This benefit provides the means to decrease dose to surrounding normal tissues and organs at risk (OARs) and thus has the theoretical potential to decrease the rates of both acute (eg, wound complications) and late complications (eg, fibrosis, joint stiffness, lymphedema, fracture). Although proton therapy has been used in the management of sarcoma for several decades, limited data exist regarding the dosimetric advantages and potential benefits of modern scanning beam proton therapy relative to conventional photon therapy modalities in attempting to reduce these acute and late toxicities [14]. In this study, we report a dosimetric comparison of photon to proton plans in adult patients with soft tissue sarcoma of the lower extremity given the increasing and evolving role of ultra-hypofractionation in cancer management.

## Materials and Methods

Institutional review board approval was obtained for this retrospective dosimetric study. Eleven consecutive patients with localized, node-negative soft tissue sarcoma of the proximal lower extremity, treated preoperatively from October 2015 to August 2020 with conventionally fractionated photon therapy at a single institution, were selected. Volumes were delineated and doses reported consistent with International Commission on Radiation Units and Measurements (ICRU) reports 50, 62, and 78. Existing computed tomography (CT) scans from the initial simulation were used to delineate gross, clinical, and planning target volumes as defined according to Radiation Therapy Oncology Group (RTOG) 0630 with the gross tumor volume (GTV) defined by T1-weighted magnetic resonance imaging (MRI) and CT [7]. The clinical target volume (CTV) was defined as a margin of at least 4 cm longitudinally and 1.5 cm radially on the GTV, in addition to any suspicious edema as seen on T2-weighted MRI, and cropped out of any uninvolved bone and nonadjacent muscle compartments. This CTV was then expanded to a planning target volume (PTV) using a 5-mm expansion. When the PTV expansion extended toward the patient surface, it was cropped 3 mm from the skin, unless there was skin involvement.

Both photon and proton treatment plans were designed to deliver a dose of 6 Gy × 5 fractions (30 Gy) to cover at least 95% of the PTV for each patient. For proton therapy, doses are reported in Gy (RBE) = 1.1 Gy. Volumetric modulated arc therapy (VMAT) and scanning beam intensity-modulated proton therapy (IMPT) planning techniques were used as detailed below. Radiation plans were deemed acceptable if they met dosimetric parameters outlined in **Table 2**. Comparative maximum, minimum, and mean doses to the adjacent OARs, in-field (1 cm above and below CTV) bone, adjacent joint, normal tissue, and body were assessed.

**Table 2.** Dose goals and constraints achieved for photon versus proton therapy.

**Target volume**	**Parameter**	**Goal or constraint**	**Proton, mean ± SD**	**Photon, mean ± SD**	***P*** **value**
GTV	V30 Gy	100%	100% ± 0%	100% ± 0%	.149
CTV	V30 Gy	>99%	99.8% ± 0.2%	99.8% ± 0.3%	.430
PTV	V30 Gy	>95%	97.9% ± 0.5%	98.4% ± 1.6%	.150
	V28.5 Gy	>100%	100% ± 0%	99.7% ± 0.3%	.012
	V 33 Gy	N/A	0% ± 0%	1.6% ± 3.1%	.049
Organ at risk					
Skin	V12 Gy	≤50%	17.3 ± 4.1%	20.15% ± 6.6%	.181
	V18 Gy	N/A	190.5 ± 75.3 cm^3^	185 ± 81.6 cm^3^	.348
	V30 Gy	N/A	28.7 ± 26.4 cm^3^	29 ± 32 cm^3^	.491
	V30 Gy	<10%	2.3% ± 2.2%	2.2% ± 2%	.402
	D 0.03 cm^3^	<33 Gy	31.3 ± 0.5 Gy	32.2 ± 1.4 Gy	.016
	Dmean	N/A	5.2 ± 1.4 Gy	6.6 ± 1.6 Gy	<.001
Strip	V12 Gy	≤10%	1.1% ± 2.4%	7.1% ± 10.3%	.013
	Dmean	N/A	0.75 ± 1.23	5.5 ± 2.7	<.001
Femur (in-field)	V30 Gy	≤50%	4.1% ± 4.9%	15.8% ± 18.3%	.009
	Dmean	N/A	10.7 ± 8.5 Gy	16.1 ± 7.7 Gy	<.001
Femoral head (N = 6)	V30 Gy	≤5 cm^3^	0.8 ± 1.8 cm^3^	2.2 ± 3.6 cm^3^	.055
	D 0.03 cm^3^	≤33 Gy	13.1 ± 14.3 Gy	18.3 ± 11.8 Gy	.016
	Dmean	N/A	2 ± 4.4 Gy	3.6 ± 6.4 Gy	.011
Joint (proximal, N = 6)	V30 Gy	≤50%	0.1% ± 0.1%	0.5% ± 1%	.074
	Dmean	N/A	1.8 ± 2.4 Gy	3.5 ± 4.4 Gy	<.001
Contralateral extremity	Dmax	<2 Gy	0.7 ± 0.4 Gy	4.3 ± 4.3 Gy	<.001
	Dmean	N/A	0.05 ± 0.04 Gy	1.1 ± 1.0 Gy	.003
Genitals (N = 4)	Dmean	N/A	0.12 ± 0.18 Gy	1.8 ± 2.4 Gy	<.001
Body − CTV	Integral dose (J)	N/A	48.3 ± 22.7	90.2 ± 34.2	<.001
Body − PTV	Integral dose (J)	N/A	37.6 ± 19.4	79.6 ± 31.9	<.001

**Abbreviations:** GTV, gross tumor volume; CTV, clinical target volume; PTV, planning target volume; N/A, not applicable; Dmean, mean dose; in-field, defined as 1 cm above and below CTV; Dmax, maximum dose; body − CTV, body minus clinical target volume; body − PTV, body minus planning target volume.

Note: N = 11, unless otherwise noted.

All photon plans were created using a VMAT technique using at least 2 arcs in Pinnacle Treatment Planning System (Philips, Amsterdam, the Netherlands) and optimization with 0.5-cm multileaf collimator thickness. Partial arcs with a minimum of 180° were chosen to reduce dose to the contralateral extremity. The monitor unit to cGy ratio was maintained at approximately 2 to avoid a highly modulated VMAT plan. For proton planning, Raystation (RaySearch Laboratories, Stockholm, Sweden) planning system version 10.A was used for all cases with the proton pencil beam plan optimized for the ProBeat Proton Therapy System (Hitachi, Tokyo, Japan). Dose calculations were performed on a 2 × 2 × 2 mm^3^ grid for both VMAT and IMPT. Proton beam arrangements were selected following the criteria that there was at least a 40° separation between each beam, at least a 45° angle between the proton beams and skin, and that the use of couch kick was avoided. Three to 4 proton beams were used based on the target volume and distance between surrounding OARs. Parallel opposed proton beams were used when the OARs could be entirely avoided. Optimal beam angles were chosen to ensure avoidance of critical OARs, ranging into critical OARs, beam path heterogeneities, and anatomy or organs that may vary in positioning. Plans were optimized using single field optimization. Gradient matching between fields was allowed when necessary to meet OAR dose constraints, for example, when the target wrapped around the bone. At least 80% of the prescription dose had to be uniformly delivered from each individual field. Up to 20% of the prescription dose could be delivered with gradient matching between 2 adjacent fields. Considerations were made for the range uncertainties from multiple sources such as energy fluctuation of the delivery machine (∼1 mm), compensator manufacturing (∼2 mm), and conversion of the CT Hounsfield number into proton stopping power (∼3.5% of distal depth of CTV). All plans were robustly optimized with a 3-mm setup uncertainty and a 3.5% range uncertainty. Circumferential irradiation of the limb was avoided to reduce the risk of lymphedema and functional deficit. Full prescription dose to skin over areas commonly traumatized (eg, knee) was also avoided. This was historically particularly relevant with proton therapy when passive scattering was used, since no skin-sparing effect was obtained [1].

Target coverage was analyzed by means of conformity index (CI) at the prescription isodose level. The Radiation Therapy Oncology Group conformity index (CIRTOG) is defined as a ratio between the volume covered by the reference isodose (95% prescription dose) and the target volume designated PTV:



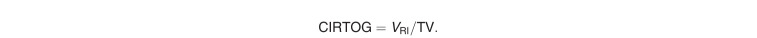




The lesion coverage volume factor (CVF) is defined by the following ratio:



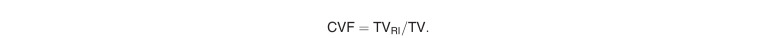




Here, V_RI_ is reference isodose volume, TV is the PTV volume, and TV_RI_ is the PTV volume covered by the reference isodose. The CIRTOG and CVF do not evaluate the irradiated volumes of normal tissues adjacent to the target especially for the volumes irradiated lower than the reference isodose (95% prescription).

We assessed the integral dose of normal tissue [body minus CTV (body − CTV) and body minus PTV (body − PTV)]. The normal tissue accounts for the whole patient body available subtracted by the CTV or PTV. The integral dose is the volume integral of the dose deposited inside that volume [15]. The normal tissue is assumed to have a tissue density of 1g/cm^3^ for the integral dose calculation.

The dose homogeneity and heterogeneity are evaluated by means of heterogeneity indices (HIs) to assess the dose heterogeneity inside the target volume. Owing to dose calculation uncertainty (0.5%) of Monte-Carlo, D_2%_, D_5%_, D_95%_, and D_98%_ are used in the following homogeneity index calculation instead of the maximum dose and minimum dose. There are 2 HI formulas used:



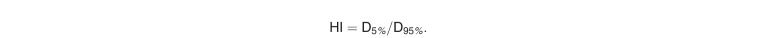





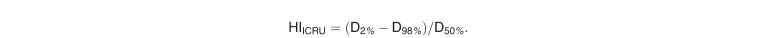




Here, D_2%_ is the near-maximum dose that covered 2% target volume, and D_98%_ is the near-minimum dose that covered 98% target volume.

Data analysis was performed using MATLAB (MathWorks, Natick, Massachusetts) and Excel (Microsoft, Redmond, Washington). Paired *t* tests were used to compare target coverage and OAR constraints for both techniques, and *P* < .05 was used to determine statistical significance.

## Results

Median age at time of treatment of was 67 years (range, 28-91 years). Seven patients (64%) were female. The soft tissue sarcoma subtypes were dedifferentiated liposarcoma (36%), myxoid liposarcoma (27%), myxofibrosarcoma (9%), undifferentiated pleomorphic sarcoma (9%), and other (18%). Median tumor size was 12 cm (range, 5.1-16.3 cm). The most common tumor stage was T2 (64%), followed by T3 (18%) and T4 (18%).

A minimum 99% CTV coverage and all OAR dose constraints were achieved for all plans, which are provided in **Table 2**. **Figure 1** shows the comparative dose-volume histogram for the target volumes and OARs. Comparable coverage of target volumes was seen between proton and photon plans. The mean CTV V99 was 100% (range, 99.6%-100%) for IMPT and 99.8% for VMAT (range, 99.8%-100%). As shown in **Table 2** and **Figure 1**, IMPT plans allowed statistically significant sparing in comparison with VMAT plans for all OARs assessed, including femur, femoral head, contralateral extremity, joint, and genitals. Mean doses to the femur (10.7 ± 8.45 vs 16.1 ± 7.7 GyRBE), femoral head (2.00 ± 4.43 vs 3.64 ± 6.38 GyRBE), and proximal joint (1.8 ± 2.4 vs 3.5 ± 4.4 GyRBE) were all significantly lower with IMPT than with VMAT plans (all *P* < .05).

**Figure 1.**
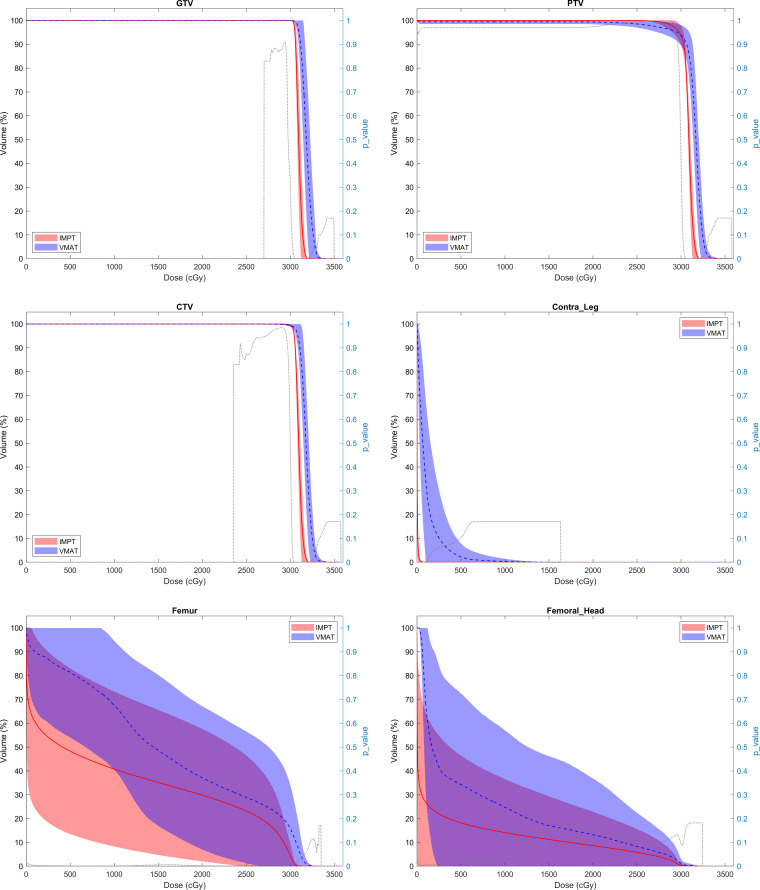
Dose-volume histogram comparison of photon vs proton therapy for target volumes and adjacent normal tissues.

**Figure 1.**
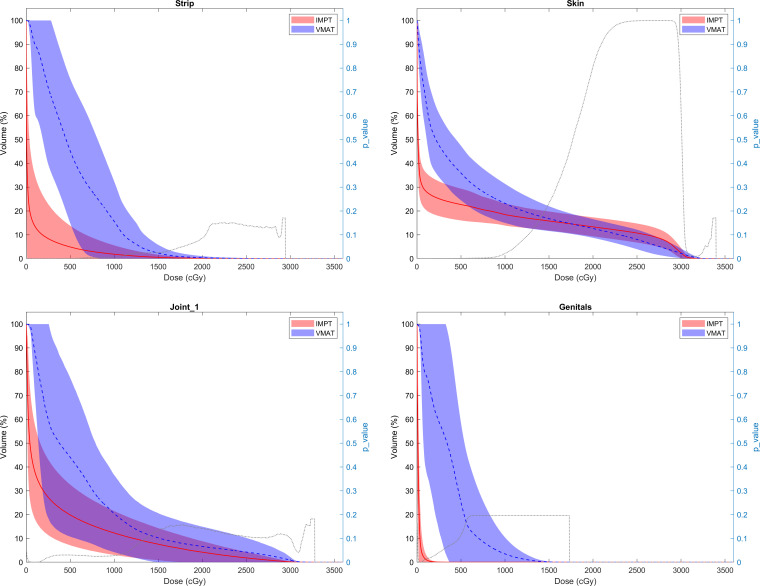
Continued.

**Figure 2.**
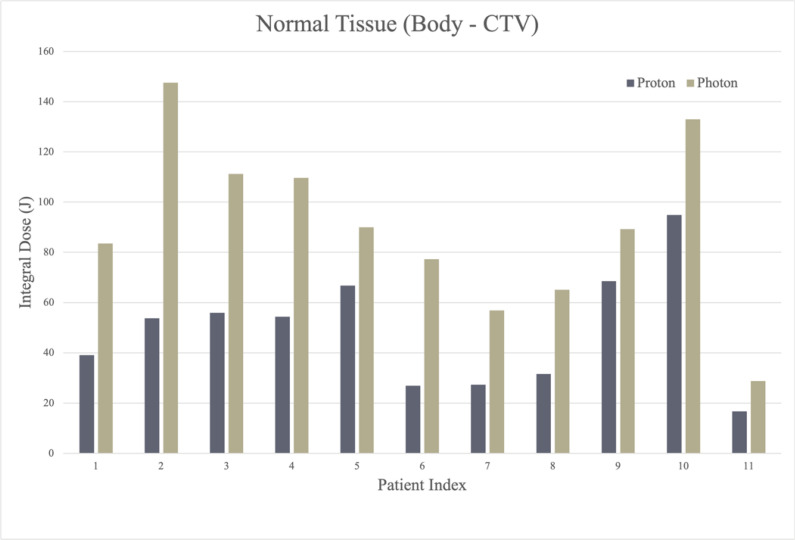
Comparative integral dose case series for photon versus scanning beam proton therapy.

Integral dose was calculated for all IMPT and VMAT plans, as provided in **Figure 2**. Integral dose decreased by an average of 97.2% ± 49.5% with IMPT compared with VMAT. The average dose to body-CTV was 148.7 ± 62.2 Gy (RBE) with IMPT and 353.9 ± 156.0 Gy with VMAT (*P* < .001). The average dose to body-PTV was 116.5 ± 50.7 Gy (RBE) for IMPT and 316.0 ± 143.8 Gy for VMAT (*P* < .001). Integral dose to the normal tissues (body-CTV) was always greater with photon plans and ranged from 1.3 to 2.9 times greater than proton plans. The mean integral dose for photon plans was 2 times greater (1.97 ± 0.52 J) for photon (90.2 ± 34.2 J) compared with proton (48.3 ± 22.7 J) plans, (*P* ≤ .001).

The CI and CVF are shown in **Table 3**. The CVF and HI were significantly better with IMPT. Proton plans also demonstrated smaller standard deviations for both the CI and CVF. IMPT plans were more homogenous, as indicated by the smaller values in both heterogeneity indices. For 2 cases (**Table 4**, cases 7 and 11), VMAT could not achieve the desired heterogeneity.

**Table 3.** CI and CVF of photon versus proton therapy.

Case index	CI^a^		CVF^b^	
	VMAT	IMPT	VMAT	IMPT
1	1.06	1.12	0.95	1.00
2	1.23	1.14	0.97	0.99
3	1.17	1.17	0.97	0.99
4	1.20	1.20	0.99	1.00
5	1.10	1.07	0.97	0.99
6	1.16	1.16	0.98	0.99
7	1.29	1.07	0.93	0.95
8	1.04	1.14	0.94	1.00
9	1.54	1.22	0.99	1.00
10	1.41	1.26	1.00	1.00
11	1.09	1.07	0.88	0.95
Mean ± SD	1.21 ± 0.15	1.15 ± 0.06	0.96 ± 0.03	0.99 ± 0.02

**Abbreviations:** CI, conformity index; CVF, coverage volume factor; VMAT, volumetric modulated arc therapy; IMPT, intensity-modulated proton therapy; PTV, planning target volume.

a CI = Volume of 95% prescription/volume of PTV; *P* = .117.

b CVF = PTV volume covered by 95% prescription/volume of PTV; *P* = .022.

**Table 4.** Heterogeneity indices for photon versus proton therapy.

Case index	HI = D_5%_/D_95%_ (%)^a^		HI = D_2%_ − D_98%_/D_50%_ (%)^b^	
	VMAT	IMPT	VMAT	IMPT
1	1.13	1.07	23.3	7.98
2	1.09	1.07	18.7	8.69
3	1.08	1.07	16.1	8.94
4	1.08	1.04	10.1	5.68
5	1.10	1.05	18.2	7.30
6	1.05	1.04	11.7	7.12
7	1.21	1.10	108.6	13.3
8	1.16	1.04	25.0	5.03
9	1.06	1.04	10.9	4.74
10	1.05	1.03	6.95	3.87
11	1.28	1.12	106.6	15.0
Mean ± SD	1.12 ± 0.07	1.06 ± 0.03	32.4 ± 37.6	7.96 ± 3.48

**Abbreviations:** HI, heterogeneity index; VMAT, volumetric modulated arc therapy; IMPT, intensity-modulated proton therapy.

b *P* = .029.

a *P* = .017.

## Discussion

In this novel study evaluating dosimetric differences between scanning beam proton therapy and photon therapy for the ultra-hypofractionated preoperative treatment of soft tissue sarcoma, we found that proton therapy provided comparable target coverage while significantly reducing the dose to the surrounding organs at risk and integral dose to the body compared with photon therapy. The **Supplemental Figure** shows a representative-dose color wash of proton (top row) versus photon (middle row) therapy including the excess-radiation dose difference (bottom row). This dose reduction to normal tissues may have the potential to reduce the incidence of postoperative wound healing and other acute and late complications such as bone fracture and lymphedema. This is of particular interest in tumors of the proximal lower extremity given that such tumors have been shown to have the highest rates of wound healing complications after preoperative RT [8]. These findings are consistent with prior studies evaluating the use of proton therapy for sarcoma, which demonstrated several potential benefits for use of protons in this setting, including the ability to dose escalate in target volumes while keeping rates of grade >3 toxicities low [16]; to decrease the dose to adjacent normal tissues [17]; and to provide a potentially more favorable toxicity profile versus conventional photon therapy in the setting of reirradiation [18,19].

There is growing evidence of the benefits of an ultra-hypofractionated approach for soft tissue sarcomas. In a single institution phase II study, Kalbasi et al [10] found that 5-day, ultra-hypofractionated, preoperative photon therapy in extremity and truncal soft tissue sarcoma had a favorable radiation toxicity profile at a median follow-up of 29 months and an acceptable rate of major wound complications. Additionally, the institution saw an increase in the number of patients treated with the ultra-hypofractionated course, suggesting that this shorter treatment course could logistically improve RT use and access to care [10]. Hypofractionated radiation therapy can reduce financial toxicities for patients compared with conventional, prolonged treatment regimens. Traditional radiation therapy regimens require daily treatment over the course of weeks, which can affect patients financially and be psychosocially burdensome. For patients who may travel out of their home cities for care at large centers, housing and other logistical costs could be significantly decreased with a shorter treatment duration. The shorter duration may be particularly effective given the limited availability of proton centers in the United States and globally. Five-day treatment courses with proton therapy have the potential to provide this same benefit while further enhancing the therapeutic ratio. The lower α/β of soft tissue sarcoma and normal tissues suggests potential benefits in tumor control and in further reducing treatment toxicities in spared tissues relative to photon therapy.

While a near-term goal would be to reduce the acute toxicities and postoperative wound complications, an additional long-term benefit of proton therapy would include reducing the risk of secondary malignancies. Our data suggest a significant reduction in integral dose for the patients in our cohort, some of whom were as young as 28 years old. Prior modeling studies in various disease sites have estimated significant reductions in the risk of secondary malignancies for patients undergoing proton therapy compared with photon therapy [20, 21]. A recent National Cancer Database analysis of over 450 000 adult and pediatric patients with a first cancer diagnosis between 2004 and 2015 was consistent with these modeling reports, showing that proton therapy had a lower risk of second cancers in comparison with IMRT (adjusted odds ratio, 0.31; 95% confidence interval, 0.26-0.36; *P* < .0001) [22].

There is limited evidence in the literature regarding skin toxicity in patients with soft tissue sarcoma, which has historically been a potential concern with proton therapy and, in particular, passive scattering techniques. Skin toxicity can play an important role in treatment planning and delivery, as acute toxicities can cause interruptions in treatment courses, and late skin toxicities can affect quality of life. A recent retrospective analysis of 79 pediatric and young adult patients treated with conventionally fractionated, nonpalliative radiotherapy with concurrent chemotherapy found no clinically meaningful differences between photon and proton therapy in acute and late radiation-induced skin toxicity [23]. This study also found V30 Gy to be a predictor of skin toxicities in patients treated with either modality. Based on these findings, we adapted the V18 Gy constraint for our analysis as a radiobiological equivalent for hypofractionation to address the potential concern for increased skin toxicity with proton therapy. Reassuringly, our results found no significant differences in the skin V30 Gy, V18 Gy, or V12 Gy between photon and proton therapy, with proton plans providing significantly lower maximum and mean skin doses than photon plans. This suggests that proton therapy can potentially provide similar and acceptable rates of skin toxicity in patients treated with 5-day preoperative proton therapy. Furthermore, the more modern pencil beam scanning technique improves skin dose compared with previous passively scattered proton techniques in which skin dose was more difficult to reduce.

A limitation of this study is the small number of patients, limiting representativeness in this already rare disease entity. Furthermore, unclear systematic differences may exist between photon RT and proton therapy algorithms, which could make the comparison of dose metrics between techniques potentially biased.^23^

In conclusion, proton therapy maintained target coverage while significantly reducing dose to the surrounding OARs and integral dose compared with photon therapy, while also showing better conformity and heterogeneity indices. Further clinical investigation is warranted to validate these dosimetric findings and potential clinical benefit in the management of adult soft tissue sarcoma and is being investigated at our institution in the form of a prospective trial.
