# Supplementary material for: Comparing Ultra-hypofractionated Proton versus Photon Therapy in Extremity Soft Tissue Sarcoma
Source: Int J Part Ther. 2023 Jan 16;9(3):30–9. doi: 10.14338/IJPT-22-00022.1 (PMC9875823; doi:10.14338/IJPT-22-00022.1)

Supplemental Figure. Representative dose color wash of ultrahypofractionated, pre-operative proton (top row) versus photon (middle row) therapy including the excess radiation dose difference (bottom row).

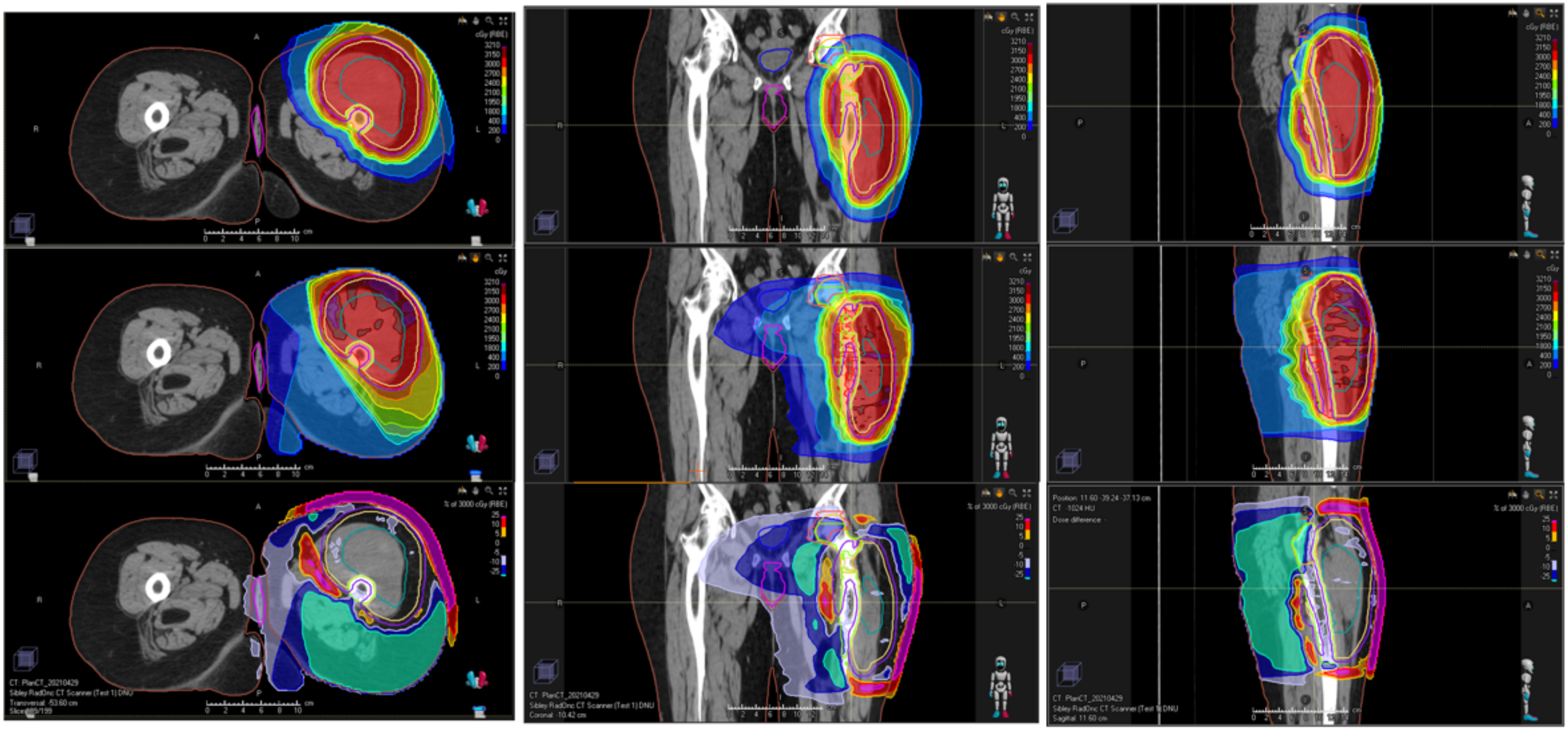

Supplement: Supplementary file 1 [file ijpt-09-03-06_s01.pdf]
